# Molecular gene signature and prognosis of non-small cell lung cancer

**DOI:** 10.18632/oncotarget.10622

**Published:** 2016-07-16

**Authors:** Poyin Huang, Chiou-Ling Cheng, Ya-Hsuan Chang, Chia-Hsin Liu, Yi-Chiung Hsu, Jin-Shing Chen, Gee-Chen Chang, Bing-Ching Ho, Kang-Yi Su, Hsuan-Yu Chen, Sung-Liang Yu

**Affiliations:** ^1^ Department of Neurology, Kaohsiung Municipal Hsiao-Kang Hospital, Kaohsiung Medical University, Kaohsiung, Taiwan; ^2^ Department of Neurology, Kaohsiung Medical University Hospital, Kaohsiung Medical University, Kaohsiung, Taiwan; ^3^ Ph.D. Program in Translational Medicine, Kaohsiung Medical University and Academia Sinica, Taiwan; ^4^ Department of Neurology, Faculty of Medicine, College of Medicine, Kaohsiung Medical University, Kaohsiung, Taiwan; ^5^ Department of Clinical Laboratory Sciences and Medical Biotechnology, College of Medicine, National Taiwan University, Taipei, Taiwan; ^6^ Institute of Statistical Science, Academia Sinica, Taipei, Taiwan; ^7^ Department of Traumatology, National Taiwan University Hospital, Taipei, Taiwan; ^8^ Faculty of Medicine, School of Medicine, National Yang-Ming University, Taipei, Taiwan; ^9^ Division of Chest Medicine, Department of Internal Medicine, Taichung Veterans General Hospital, Taichung, Taiwan; ^10^ Comprehensive Cancer Center, Taichung Veterans General Hospital, Taichung, Taiwan; ^11^ Center of Genomic Medicine, National Taiwan University, Taipei, Taiwan; ^12^ Center for Optoelectronic Biomedicine, College of Medicine, National Taiwan University, Taipei, Taiwan; ^13^ Graduate Institute of Pathology, College of Medicine, National Taiwan University, Taipei, Taiwan; ^14^ Department of Laboratory Medicine, National Taiwan University Hospital, Taipei, Taiwan

**Keywords:** non–small cell lung cancer, TaqMan Low-Density Array, risk score, gene signature, prognosis

## Abstract

The current staging system for non–small cell lung cancer (NSCLC) is inadequate for predicting outcome. Risk score, a linear combination of the values for the expression of each gene multiplied by a weighting value which was estimated from univariate Cox proportional hazard regression, can be useful. The aim of this study is to analyze survival-related genes with TaqMan Low-Density Array (TLDA) and risk score to explore gene-signature in lung cancer. A total of 96 NSCLC specimens were collected and randomly assigned to a training (*n* = 48) or a testing cohort (*n* = 48). A panel of 219 survival-associated genes from published studies were used to develop a 6-gene risk score. The risk score was used to classify patients into high or low-risk signature and survival analysis was performed. Cox models were used to evaluate independent prognostic factors. A 6-gene signature including ABCC4, ADRBK2, KLHL23, PDS5A, UHRF1 and ZNF551 was identified. The risk score in both training (HR = 3.14, 95% CI: 1.14–8.67, *p* = 0.03) and testing cohorts (HR = 5.42, 95% CI: 1.56–18.84, *p* = 0.01) was the independent prognostic factor. In merged public datasets including GSE50081, GSE30219, GSE31210, GSE19188, GSE37745, GSE3141 and GSE31908, the risk score (HR = 1.50, 95% CI: 1.25–1.80, *p* < 0.0001) was also the independent prognostic factor. The risk score generated from expression of a small number of genes did perform well in predicting overall survival and may be useful in routine clinical practice.

## INTRODUCTION

Lung cancer, predominantly non–small-cell lung cancer (NSCLC), is the most common cause of cancer deaths worldwide [1]. The relapse rate among patients with early-stage NSCLC is 40% within 5 years after potentially curative treatment [2]. The current TNM staging system provides guidance to the arrangement of initial treatments [3]. It is also a valuable indicator for predicting patient survival. However, for lung cancer, this system does not perform as well as in other cancers since 40% of patients with early stage in lung cancer relapsed within five years [4]. Thus, the current staging system for NSCLC is inadequate for predicting the outcome of treatment.

Results of molecular research may improve the management of patients. Advances in genomics and proteomics have generated many candidate markers with potential clinical value [5]. Gene expression profiling by microarray or real-time RT-PCR can be useful tools for identifying genes involved in the etiology or progression of cancer [5–17], as well as many other diseases [18–23]. The molecular signature can provide additional information for treatment decision. For example, the molecular status of the epidermal growth factor receptor (EGFR) in lung cancer [24–25] has been shown to have influence on the prognosis of patients and may indicate the introduction of alternative therapies. The combined use of TNM staging system and gene signature may enhance the prediction accuracy of survival and help avoid unnecessary treatment. Thus gene signature can provide extra information beyond the staging system. Furthermore, the gene signature also can be used to identify patients who are responsive to chemotherapy. After stratification by excision repair cross-complementation group 1 (ERCC1) expression, patients who received cisplatin-based chemotherapy had prolonged survival only for those with negative ERCC1 status but not for those with positive ERCC1 status [26–27]. A similar result was found in the study of gefinitib in lung cancer [28]. Lung adenocarcinoma with EGFR activating mutations had a higher response rate.

The genes that significantly correlated with clinical outcomes can be used to derive a predictive model for patients' survival. There were many algorithms available in the literature for predictive model developments [10, 29]. However, the complex structures of most algorithms or models have substantially reduced their potential in clinical application. But these limitations of microarray do not wipe out completely its benefits in exploratory studies wherein it is used as a screening tool. In contrast, RT-PCR is a faster and more stable assay, which is more suitable for clinical practice [30–33]. Although many studies reported that one single gene could predict clinical outcomes successfully, these findings need to be validated in more validation cohorts. A single gene may exhibit a strong association with clinical outcome before it was used as a classifier [34, 35]. In order for a single-gene-based classifier to reach a high accuracy level (i.e. sensitivity = 0.8 and specificity = 0.9), the odds ratio of the predictor gene needs to be as high as 228 [34, 35]. A combination of several potential genes may help to surpass this limitation. The small effect of each gene can be cumulated to improve the overall predictive power. For instance, the risk score, a linear combination of weighted gene expression, can be useful if properly constructed [4, 7, 31, 32, 36].

Until today, many studies demonstrate the gene signatures for survival in lung cancer by using public database [33, 37, 38]. However, there is no consistent gene-signature generated from the current studies. Hence, the aim of our study is to analyze the huge published survival-related genes measured by TaqMan Low-Density Array and risk score to explore the robust lung cancer gene-signature in lung cancer. The identification of these gene signatures will help scientists to develop not only robust gene signature for prognosis prediction but also the potential druggable targets for lung cancer.

## RESULTS

**Figure 1:**
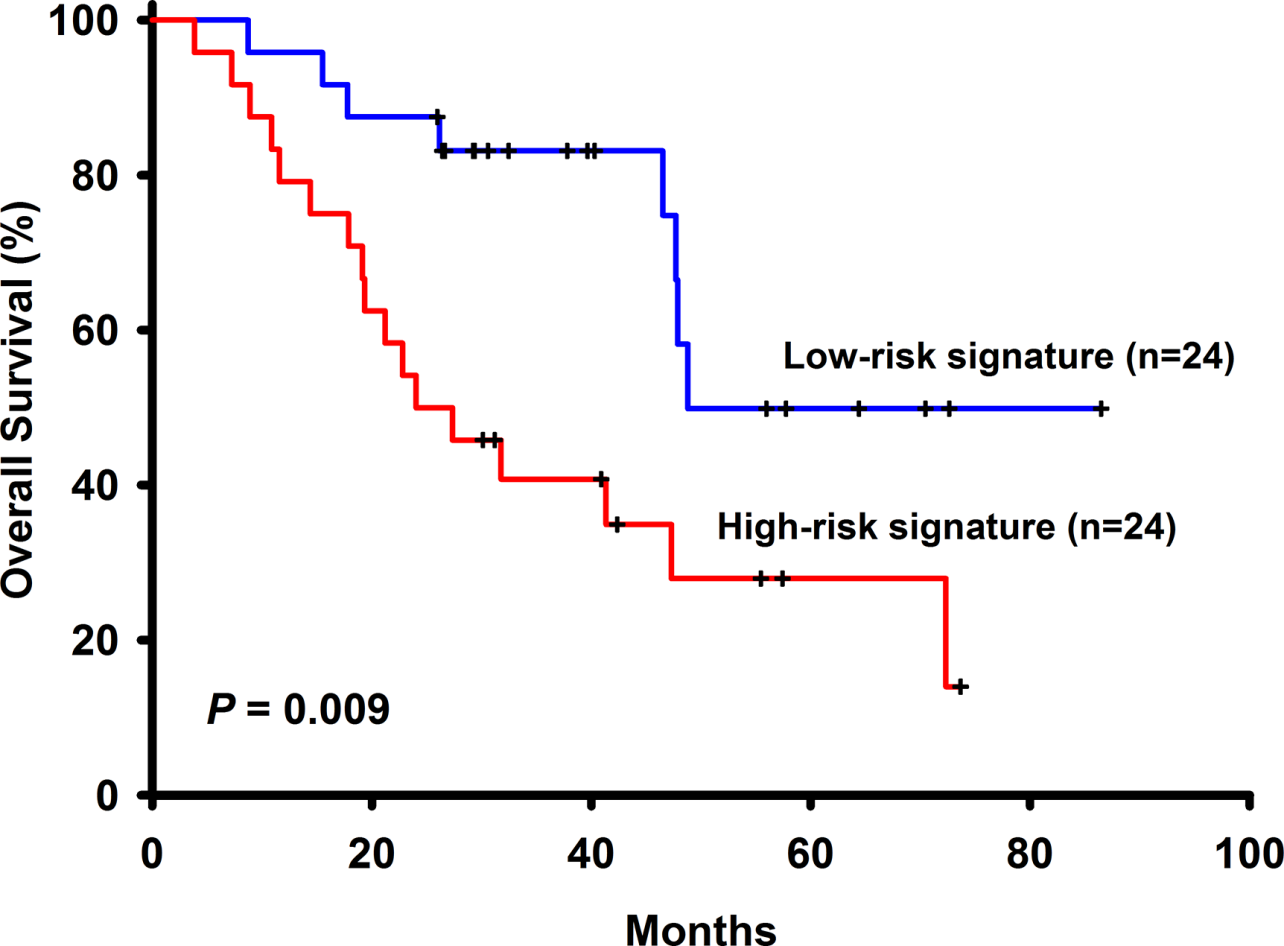
The survival analysis of the 6-gene signautre in the training cohort

**Figure 2:**
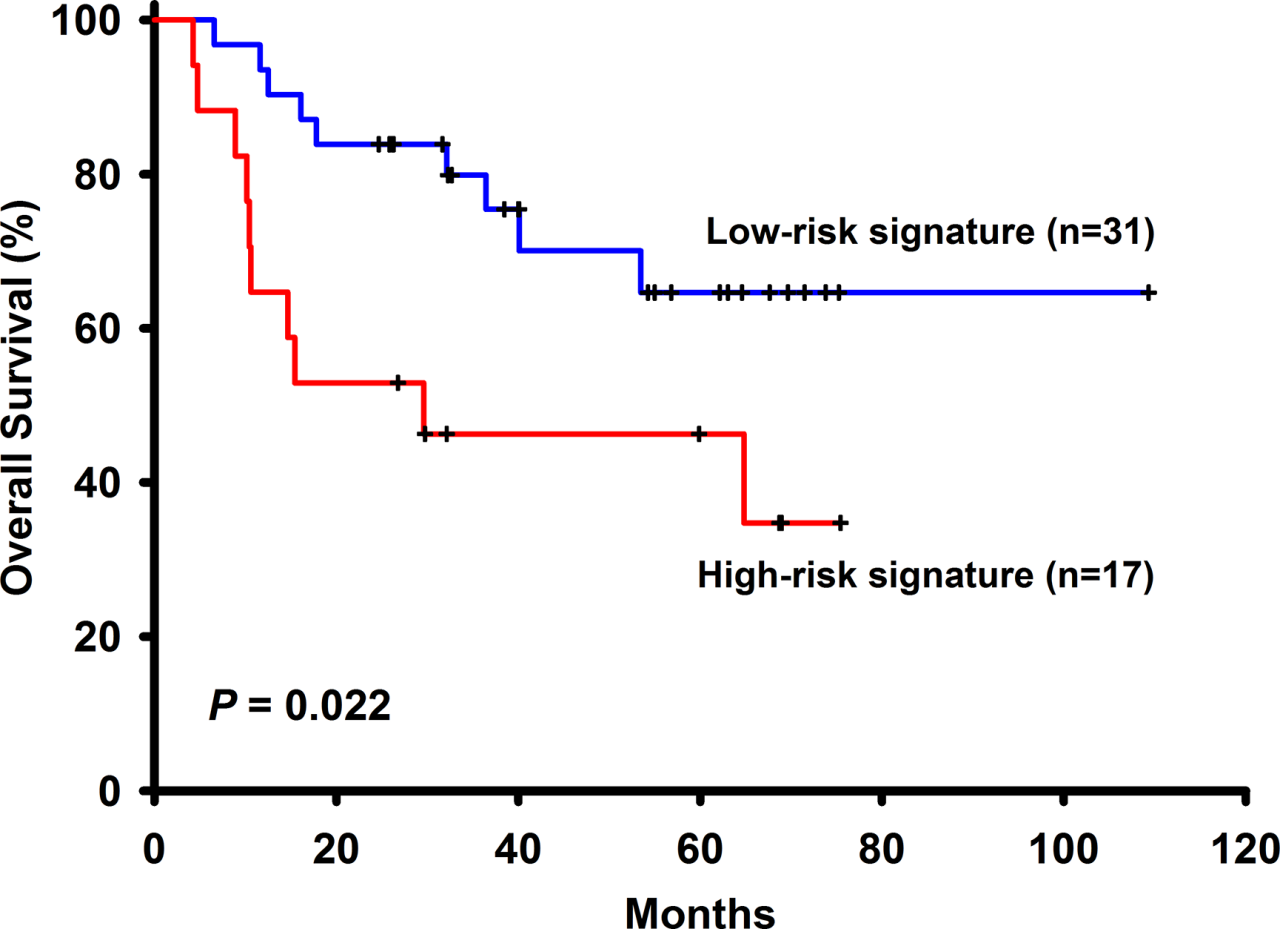
The survival analysis of the 6-gene signautre in the testing cohort

Among the 96 NSCLC patients, 50 (52.08%) have adenocarcinoma and 46 (47.92%) have squamous cell carcinoma. There is one missing data in stage, thus 58 (61.05%) patients have stage I, 14 (14.74%) have stage II and 23 (24.21%) have stage III NSCLC. These patients had not received adjuvant chemotherapy with a median follow-up time of 32.13 months (range 3.83 to 109.33). Other basic characteristics of the patients were shown in Table 1. Among the 258 selected genes, 24 (LOC158381, ALPPL2, C3orf45, CALCA, CASR, CCKBR, CYP3A4, CYP3A43, DEFA6, FEV, FGF4, FLJ16124, FLJ21511, IGLL1, IL11, LCT, MEP1B, PDIA2, PTBP1, SLC1A7, SLC26A3, TBC1D29, MYCNOS, C5orf24) of them were excluded from the panel because more than half of the patients (> 48 patients) have undetermined gene expression level. Univariate Cox regression analysis was performed to find genes associated with survival and a total of 6 genes such as ABCC4, ADRBK2, KLHL23, PDS5A, UHRF1 and ZNF551 were found to have significant associations with overall survival (Table 2) Pathways analyses showed that there were no interactions between these genes and only ADRBK2 was shown to be significantly involved in Cardiac β-adrenergic Signaling pathway. These 6 genes were not involved in the same pathway thus collinearity should not be a concern. For each patient, a risk-score according to a linear combination of the expression level of these 6 genes was used to classify patients into high or low risk signature. In the training group (*n* = 48), 24 patients are low risk and 24 patients are high risk. In univariate Cox model, risk score classifying patients into high or low risk signature (HR = 2.94, 95% CI: 1.26–6.85, *p*−0.01) was associated with patient survivals. In multivariate Cox model, risk score classifying patients into high or low risk signature (adjusted HR = 3.14, 95% CI: 1.14–8.67, *p* = 0.03), along with NSCLC stage (adjusted HR = 4.66, 95% CI: 1.51–14.39, *p* = 0.01), are both independent prognostic factors (Table 3 and Figure 1). In the testing group (*n* = 48), 31 patients are low risk and 17 patients are high risk. Identical to the result in the training group, in univariate Cox model, risk score classifying patients into high or low risk signature (HR = 2.77, 95% CI:1.12–6.85, *p* = 0.03) was associated with patient survivals. In multivariate Cox model, risk score classifying patients into high or low risk signature (adjusted HR = 5.42, 95% CI: 1.56–18.84, *p* = 0.01), along with NSCLC stage (adjusted HR = 11.18, 95% CI: 3.43–36.40, *p* < 0.001), are both independent prognostic factors (Figure 2).

**Table 1:** Basic clinical characteristics of the study population

	ALL (*n* = 96)		Training group (*n* = 48)		Testing group (*n* = 48)		*p* – value
Gender							1.000
Female	22	22.92	11	22.92	11	22.92	
Male	74	77.08	37	77.08	37	77.08	
Histology							1.000
SCC	46	47.92	23	47.92	23	47.92	
Adenocarcinoma	50	52.08	25	52.08	25	52.08	
Stage							0.339
I/II	72	75.79	38	80.85	34	70.83	
III	23	24.21	9	19.15	14	29.17	
Stage							0.374
I	58	61.05	32	68.09	26	54.17	
II	14	14.74	6	12.77	8	16.67	
III	23	24.21	9	19.15	14	29.17	
Age							0.207
mean, sd	67.11	10.54	68.47	10.95	65.75	10.04	

Data are presented as mean ± standard deviation or n (%);*p* value by χ^2^ or two-tailed *t* test. One patient has missing data in stage (*n* = 95).

**Table 2:** 6-gene signature identified from Cox model of training group (*n* = 48)

Variable	coefficient	HR	HR, 95% CI		*p* - value
ABCC4-Hs00988734_m1	−0.22096	0.802	0.693	0.927	0.0029
ADRBK2-Hs01007260_m1	−0.52732	0.59	0.408	0.853	0.0051
KLHL23;PHOSPHO2-Hs00376354_m1	−0.64501	0.525	0.335	0.822	0.0049
PDS5A-Hs00374857_m1	−0.62813	0.534	0.361	0.79	0.0017
UHRF1-Hs01086727_m1	0.45151	1.571	1.129	2.185	0.0073
ZNF551-Hs00292939_m1	−0.28384	0.753	0.621	0.912	0.0037

**Table 3:** Multivariate Cox model of training, testing and validation cohorts

Variable	Hazard Ratio (95% CI)	*P* Value
**Training cohort**		
High-risk six-gene signature	3.14 (1.14–8.67)	0.027
Male	1.74 (0.39–7.82)	0.47
Older age	1.02 (0.97–1.07)	0.43
Adenocarcinoma	2.65 (0.95–7.43)	0.06
Tumor stage III	4.66 (1.51–14.39)	0.01
**Testing cohort**		
High-risk six-gene signature	5.42 (1.56–18.84)	0.008
Male	7.23 (0.81–64.97)	0.08
Older age	1.01 (0.94–1.08)	0.83
Adenocarcinoma	0.97 (0.35–2.70)	0.95
Tumor stage III	11.18 (3.43–36.40)	< .0001
**Validation cohort**		
High-risk six-gene signature	1.50 (1.25–1.80)	< .0001
Adenocarcinoma	0.65 (0.54–0.78)	< .0001
Male	1.43 (1.17–1.74)	0.0005

**Figure 3:**
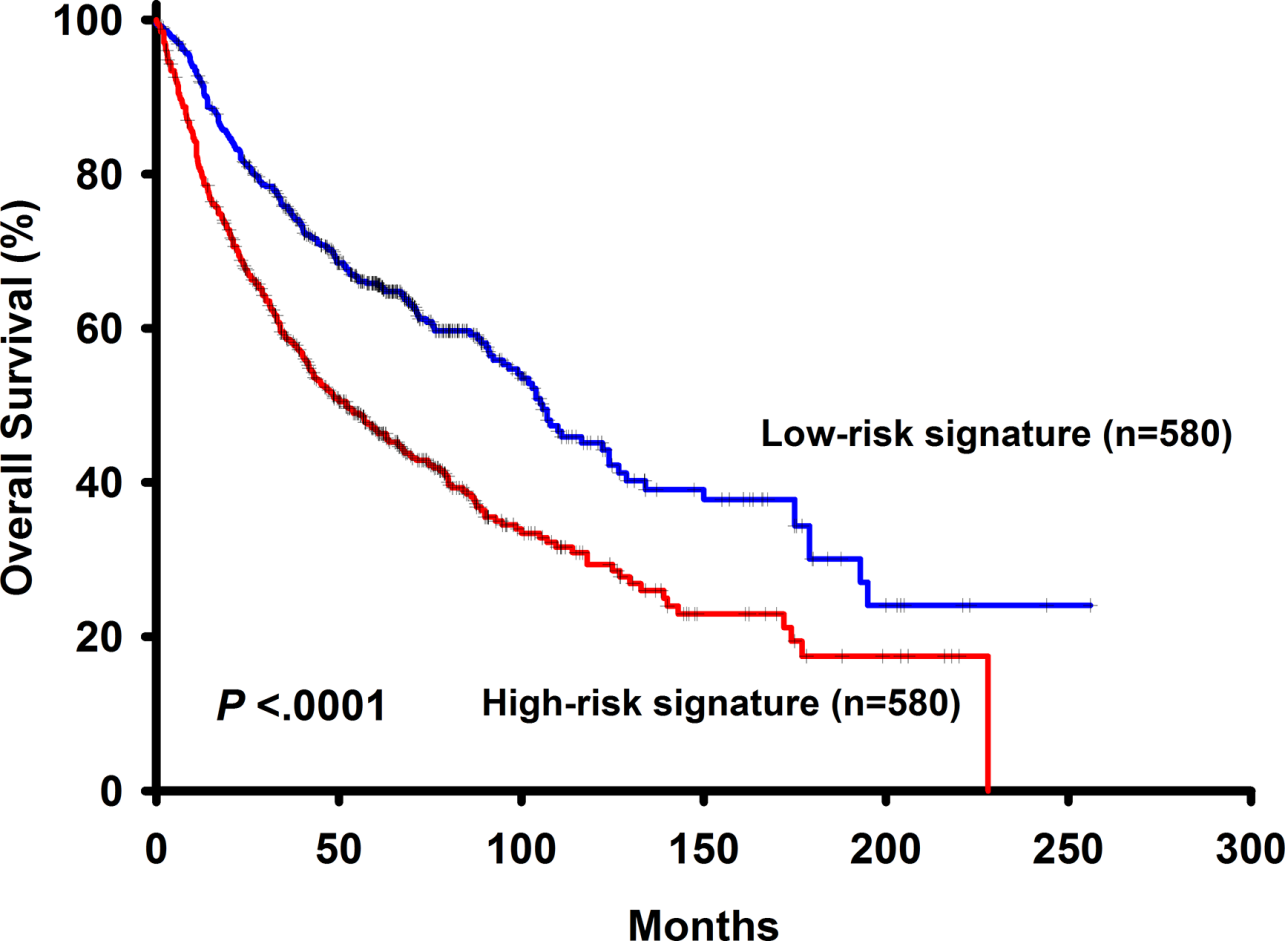
The survival analysis of the 6-gene signautre in the validation dataset

To further validate our findings, the risk score derived from 6 genes associated with overall survival was applied directly to merged public datasets including GSE50081, GSE30219, GSE31210, GSE19188, GSE37745, GSE3141 and GSE31908 GSE3141. The basic characteristics of the validation cohort were shown in Supplementary Table 1. In this dataset, result of the survival analysis showed that patients with high risk signature had shorter overall survival (*p* < 0.0001) (Figure 3). In univariate Cox model, the 6-gene risk signature was a risk factor of patients' survival (HR = 1.74, 95% CI: 1.47–2.05, *p* < 0.0001). In multivariate Cox model, the 6-gene risk signature (adjusted HR = 1.50, 95% CI: 1.25–1.80, *p* < 0.0001), histology (adjusted HR = 0.65, 95% CI: 0.54–0.78, *p* < 0.0001) and gender (adjusted HR = 1.43, 95% CI: 1.17–1.74, *p* = 0.0005) are independent prognostic factors. Overall, the risk score, based on a linear combination of the expression level of 6 genes, which classified patients into high or low risk signature, is consistently an independent prognostic factor associated with NSCLC patient survivals.

## DISCUSSION

NSCLC is a heterogeneous disease resulting from multiple somatic mutations. Due to the complexity, it is less likely that a single gene expression pattern could be effectively used to predict the clinical course and outcome of NSCLC for all patients [15]. Instead, multiple sets of gene expression patterns may exist in tumors. Thus, it is believed that multiple sets of gene expression signatures that can be used for outcome prediction exist in NSCLC [32–33]. Despite the breakthrough in next-generation sequencing technology, microarray technologies are still useful platforms for biological exploration. Lung cancer has been among the earliest and most intensely studied diseases using microarray platforms [39]. Two very recent studies have used microarray technologies to derive a robust prognostic gene expression signature for early stage lung adenocarcinoma [40] and identify a 17 gene expression signature that distinguishes lymphangiogenic from non-lymphangiogenic NSCLC cell lines [41]. Molecular signatures help to reveal the biologic spectrum of lung cancers, throw light on the pathogenetic alterations in gene expressions and cellular pathways, identify prognostic and predictive gene signatures, customize therapies, identify new therapeutic targets and evaluate new drugs [39]. The small effect of each gene can be cumulated and a combination of several potential genes may help to improve the overall predictive power. In this study, we use the risk score algorithm to combine several potential genes to surpass the limitation of using a single gene expression pattern to predict NSCLC outcome.

Adenocarcinomas and squamous carcinomas are distinct disease entities with different gene expression patterns, thus using independent prognostic signatures for squamous carcinomas and adenocarcinomas should be more biologically significant and less affected by genetic heterogeneities [32, 37]. Generally, the gene signature selected from one cell type is predictive for that specific cell type. Whether gene signatures from different cell types can be predictive for each other in NSCLC is still an unidentified issue, with implications as to how gene expression signatures could be translated into clinical practice. One previous study has shown that a prognostic signature may not be cell type specific and that a universal signature reflecting tumor aggressiveness and subsequent clinical outcome may exist across histologic cell types [37]. This would be of clinical importance because unified gene signatures would dramatically simplify the prognosis evaluation process for different types of carcinoma. Since a gene signature selected from adenocarcinoma or squamous cell carcinoma may be predictive for both histologic subtypes, specific prognostic signatures for NSCLC will be more attractive due to their broader applicability [37–38]. Thus, patients of squamous carcinoma and adenocarcinoma are equally represented in this study so that cancer specific, rather than histology specific signatures could be generated by our experimental method.

There are some potential clinical implications of our findings. First of all, they provide a prognostic tool. Furthermore, they might identify targets for molecular therapies and aid in directing the options of therapeutic regime. Their potential exploitation for the identification of novel therapeutic targets is directly linked to whether the gene panel is associated with lung carcinogenesis. However, expression profile analysis generally can not lead to immediate biological implications [24–25]. As might be expected, these genes, if without an established role in the pathogenesis of NSCLC, may be proved to be of no prognostic value when used individually. However, although the mechanisms by which some of these genes affect patient survival are not clarified, at least it seemed that the expression pattern of these genes might have some crucial information for NSCLC prognosis. In order to understand how these 6-signature identified in this study may influence survival in patients with NSCLC, information on the functions of the encoded proteins was obtained from the GeneCards database (http://www.genecards.org/) and described as follows: PDS5A is a cell cycle related gene. PDS5A has been shown to improve cell proliferation in G2/M phase. It may contribute to tumorigenesis by upregulating p63 and promoting cell cycle progression. PDS5A is a nuclear protein and involves in the establishment, maintenance and dissolution of sister chromatid cohesion. Altered expression levels of PDS5A have been observed in tumors of the breast, kidney, esophagus, stomach, liver and colon and in malignant gliomas [42]. ABCC4 is a protein coding gene and the protein encoded is a member of the superfamily of ATP-binding cassette transporters. These proteins transport various molecules across extra and intracellular membranes. This protein also involved in multidrug resistance. Diseases associated with ABCC4 include lung cancer and hemostasis is involved in its associated pathways. ABCC4 was highly expressed in lung cancer cell lines. ABCC4 expression was markedly downregulated in A549 and 801D cells using the RNA interference technique. Suppression of ABCC4 expression inhibited cell growth. The percentage of cells in G1 phase was increased when ABCC4 expression was suppressed. Phosphorylation of retinoblastoma protein was weakened, originating in the downregulation of ABCC4. ABCC4 mRNA was highly expressed in lung cancer tissue and lung cancer cell lines. ABCC4 is a potential target for lung cancer therapy [43]. ADRBK2 encodes the beta adrenergic receptor kinase which specifically phosphorylates the agonist occupied form of the beta adrenergic and related G protein coupled receptors. The existence of this receptor kinase may serve to desensitize synaptic receptors. Endocytosis and chemokine signaling are involved in its associated pathways. The β2-adrenergic receptor is most frequently involved in carcinogenic processes. Earlier studies have established a relation between the β2-adrenergic receptor and various characteristics of cancer including cell proliferation, apoptosis, chemotaxis, metastasis, tumor growth and angiogenesis [44]. KLHL23 has strong similarities with gene KLHL7 and not much information could be obtained regarding KLHL23. KLHL7 antibodies are associated with various types of cancer, such as ovary, rectum, colon, lung and prostate cancer. However, the function of KLHL7 is unknown [45]. UHRF1 can influence the cell cycle control mainly through the epigenetic silencing of relevant tumor suppressors. UHRF1 mRNA was overexpressed in NSCLC tumor tissues in comparison to their normal adjacent tissue. UHRF1 is a key epigenetic switch, which controls cell cycle in NSCLC through its ability to sustain the transcriptional silencing of tumor suppressor genes by maintaining their promoters in a hypermethylated status [46]. ZNF551 is a protein coding gene and may be involved in transcriptional regulation, no other information could be obtained from the literature. Further research on their potential functional role in modulating the survival of NSCLC patients is warranted [47].

In conclusion, we selected 6 genes that were associated with NSCLC patients' survival from expression data obtained by TLDA. The risk score algorithm was then successfully used to categorize patients into better and poorer prognosis since that the risk score algorithm did perform well in predicting overall survival for NSCLC in validation dataset. The result is of clinical interest that the risk score generated from expression of a relatively small number of genes [48] may be useful in routine clinical practice. Additionally, pair-samples were not available for analysis. However, since the aim of this study is to develop gene signature for prognosis prediction, the influence of not using normal tissues for analyses may be attenuated.

## MATERIALS AND METHODS

### Patients and tissue specimens

The frozen specimens of tumor tissue from 96 patients who underwent surgical resection of NSCLC at the Taichung Veterans General Hospital and the National Taiwan University Hospital were enrolled in this study Squamous carcinoma (*n* = 46) and adenocarcinoma (*n* = 50) histology are represented equally so that cancer-specific, rather than histology-specific markers may be elicited by the experimental method [46–47]. None of the patients underwent radiotherapy or chemotherapy prior to surgery. These patients had not received adjuvant chemotherapy with a median follow-up time of 32.13 months (range 3.83 to 109.33). Complete clinical information such as age, gender, stage, histological cell type, follow-up time, and survival status were collected. The present study was approved by the Ethics Committee and written informed consent was acquired from each patient.

### RNA extraction and mRNA isolation

Total RNA isolation was performed by using RNazol B reagent. Cells were lysed directly in a culture T-flask (Falcon) by adding 1.5 ml of RNazol B reagent (1 ml/100 mm^2^) to a 150 mm^2^ tissue flask, and total RNAs from the cells were obtained by using RNAzol^TM^ B to extract total RNAs and 100% isopropanol to precipitate RNAs. OligotexTM mRNA Midi Kit (QIAGEN) was used to gain mRNAs from the total RNAs mentioned above.

### Gene expression profiling

TaqMan low-density RT PCR arrays (Applied Biosystems) were designed to determine expression of the selected genes. Total RNA (0.5 μg) extracted was reverse transcribed with 200 U Superscript II RT (Invitrogen) and 250 ng random hexamers. A reaction mix containing 75 ng of cDNA and 50 μl of 2× PCR Master Mix (Euregentec) was added to a TaqMan microfluidic card. Reverse transcription and rt PCR was performed in a 7900HT RT PCR System (Applied Biosystems). The cycling program used was 50°C for 30 min, 94.5°C for 15 min, then 40 cycles of 96°C for 30 s and 59.7°C for 1 min. The expression level of each gene was measured in triplicate, and a panel of reference genes (ACTB, B2M, GAPDH, GUSB, HMBS, HPRT1, IPO8, PGK1, POLR2A, PPIA, TBP, TFRC, UBC, YWHAZ, 18S) was used. The average Ct value of each target gene was normalized against the geometric mean of the Ct values of the 15 reference genes. Relative gene expression was expressed as 2^−ΔCt^.

### Candidate gene selection

A panel of genes correlated with survival for lung cancer were collected from published studies. The including criteria of studies are journal quality, microarray study, and study of meta-analysis. A total of 258 genes (including 15 endogenous control genes, Supplementary Table 2) correlated with patients' survival published on Journal of Clinical Oncology and Journal of Clinical Investigation were selected [32–33, 37–38]. Among these 258 genes, 24 undetermined (> 48 patients) genes and 15 reference genes were excluded from further analyses. Thus, a total of 219 genes remained.

### Training and testing group

The flow chart of this study was shown in Supplementary Figure 1. The 96 specimens were randomly assigned to a training cohort (*n* = 48) or a testing cohort (*n* = 48). Using the training cohort, based on the expression level of each gene, univariate Cox model was performed. Thus, a total of 219 models were developed. In each attempt to acquire ideal gene signature, 6 genes with *p* value < 0.01 in the univariate Cox model were selected to form the risk score.

### Risk score algorithm

To investigate the effectiveness of candidate genes as a gene signature for clinical outcome prediction, a mathematical formula for survival prediction was constructed, taking into account both the strength and the positive or negative association of each gene with survival. More specifically, we assigned each patient, a risk-score according to a linear combination of the expression level of the genes, weighted by the regression coefficients derived from aforementioned univariate Cox regression analyses [7, 31]. There were 6 genes significantly had high or low risk for patient survival through univariate Cox regression analysis and the regression coefficients were as follows: ABCC4, −0.22096; ADRBK2, −0.52732; KLHL23,−0.64501; PDS5A, −0.62813; UHRF1, 0.45151; ZNF551, −0.28384 (Table 2). Then a patient's risk score was derived by a summation of each gene expression level times its corresponding coefficient. Risk score can be expressed as: −(0.22096 × ABCC4 value) − (0.52732 × ADRBK2 value) − (0.64501 × KLHL23 value) − (0.62813 × PDS5A value) + (0.45151 × UHRF1 value) − (0.28384 × ZNF551 value). The risk score was used to classify patients into high or low risk signature based on median as cut-off point. In the testing dataset, both the regression coefficients of risk score and the cut-off value derived from the training dataset were applied directly.

### Ingenuity pathway analysis

The 6 survival-associated genes were further analysed for biological function or involvement in different pathways using Ingenuity Pathway Analysis software (IPA, Igenuity Systems, USA).

### Survival analysis

The Kaplan-Meier method was used to estimate overall survival. Differences in survival between the two groups were analyzed using the log-rank test. Both univariate and multivariate Cox proportional hazard regression analyses were used to evaluate independent prognostic factors associated with patient survivals. Age, gender, histology and stage were used as covariates.

### Validation cohort

Public datasets including GSE50081, GSE30219, GSE31210, GSE19188, GSE37745, GSE3141 and GSE31908 were merged for validation analysis. These datasets used Affymetrix U133 Plus 2.0 as platform and there is a total of 1160 NSCLC patients available for analysis. The compatible probesets of acquired 6-gene signature were identified and the gene expression levels were obtained (Supplementary Table 3). Variables with more than 10% missing data were not added in the multivariate analysis.

## SUPPLEMENTARY MATERIALS FIGURE AND TABLES




