# Supplementary material for: Molecular gene signature and prognosis of non-small cell lung cancer
Source: Oncotarget. 2016 Jul 16;7(32):51898–907. doi: 10.18632/oncotarget.10622 (PMC5239522; doi:10.18632/oncotarget.10622)
Supplement: Supplementary file 1 [file oncotarget-07-51898-s001.pdf]

Molecular gene signature and prognosis of non–small cell lung cancer

Supplementary Materials

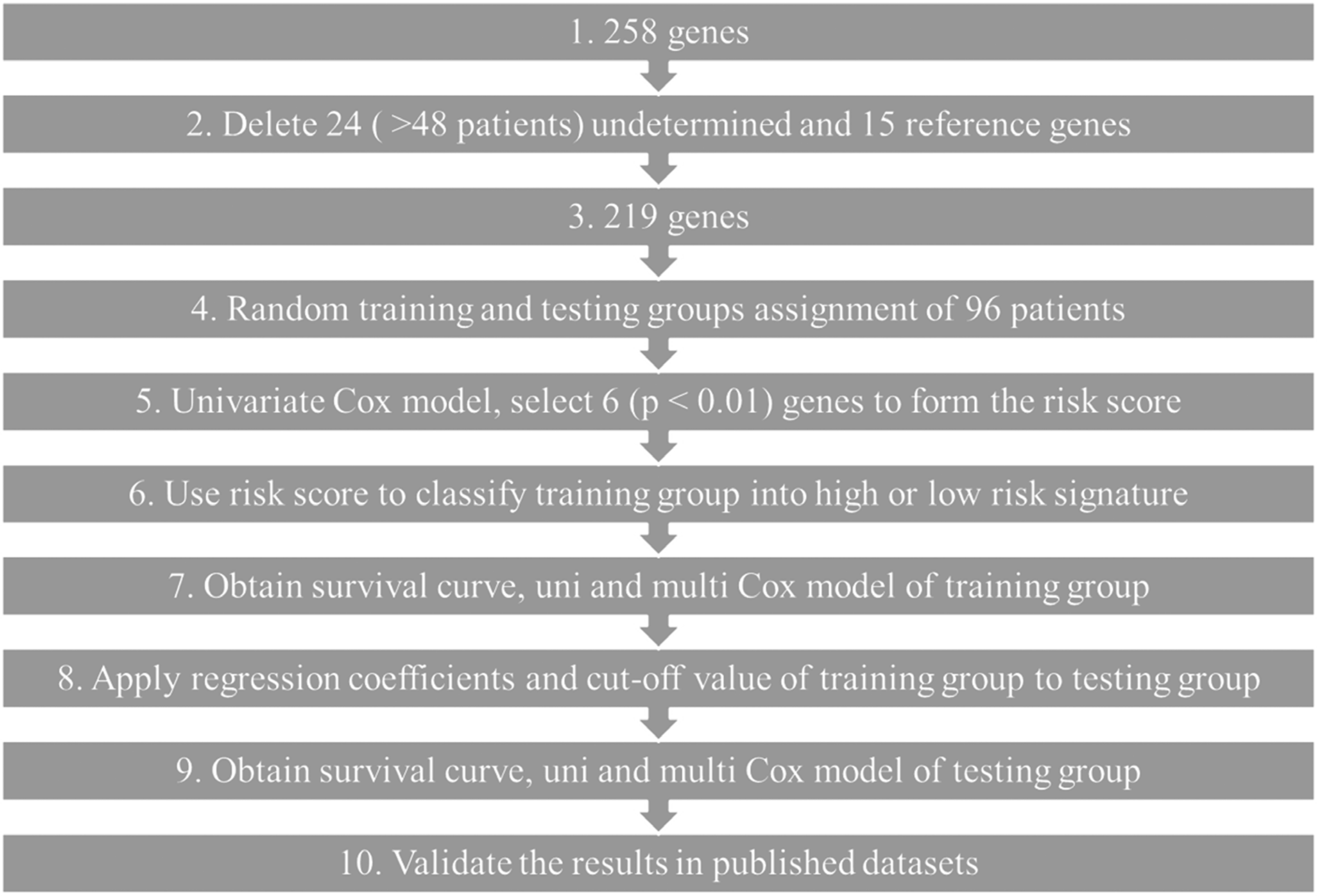

Supplementary Figure S1: The overall analytic process of candidate gene selection, risk score algorithm, survival analysis and validation.

Supplementary Table S1: Basic characteristics of the validation cohort (merged public datasets)

	ALL (*n* = 1160)		Low signature group (*n* = 580)		High signature group (*n* = 580)		*p*-value
Gender (* = 111)							<.0001
Female	389	(37.08)	228	(44.02)	161	(30.32)	
Male	660	(62.92)	290	(55.98)	370	(69.68)	
Stage (*= 193)							<.0001
1	612	(63.29)	367	(72.39)	245	(53.26)	
2	209	(21.61)	96	(18.93)	113	(24.57)	
3	131	(13.55)	42	(8.28)	89	(19.35)	
4	15	(1.55)	2	(0.39)	13	(2.83)	
Smoking (*= 703)							0.0008
No	145	(31.73)	95	(37.70)	50	(24.39)	
Yes	312	(68.27)	157	(62.30)	155	(75.61)	
EGFR mutation (* = 918)							0.0001
No	107	(44.21)	48	(34.29)	59	(57.84)	
Yes	135	(55.79)	92	(65.71)	43	(42.16)	
Histology (* = 0)							<.0001
Adenocarcinoma	690	(59.48)	420	(72.41)	270	(46.55)	
SCC	293	(25.26)	99	(17.07)	194	(33.45)	
Large cell carcinoma	110	(9.48)	28	(4.83)	82	(14.14)	
others	67	(5.78)	33	(5.69)	34	(5.86)	
Age (* = 210)							0.1762
Mean, sd	63.02 ± 10.13		62.60 ± 11.09		63.48 ± 8.97		

Data are presented as mean ± standard deviation or *n* (%); *p* value by χ^2 or two-tailed *t* test. * = missing data.

Supplementary Table S2: Genes correlated with patients' survival. See Supplementary_Table_S2

Supplementary Table S3: Probesets of acquired 6-Gene signature

Gene Name	Probeset ID
ABCC4	1554918_a_at ;1555039_a_at ;203196_at ;243928_s_at
ADRBK2	204183_s_at ;204184_s_at ;228771_at
KLHL23	213610_s_at ;217505_at ;241682_at
PDS5A	212138_at ;212140_at ;213983_s_at ;213984_at
UHRF1	225655_at
ZNF551	242992_at ;243194_at ;243195_s_at ;211721_s_at
